# Autoimmune Hepatitis: Progress from Global Immunosuppression to Personalised Regulatory T Cell Therapy

**DOI:** 10.1155/2016/7181685

**Published:** 2016-05-18

**Authors:** Nwe Ni Than, Hannah C. Jeffery, Ye H. Oo

**Affiliations:** ^1^Centre for Liver Research and NIHR Liver BRU, University of Birmingham, Edgbaston, Birmingham B15 2TT, UK; ^2^Liver and Hepatobiliary Unit, University Hospital Birmingham National Health Service Foundation Trust, Birmingham B15 2WB, UK

## Abstract

Autoimmune hepatitis (AIH) is an immune mediated liver injury. The precise aetiology of AIH is still unknown but current evidence suggests both genetic and environmental factors are involved. Breakdown in peripheral self-tolerance, and impaired functions of FOXP3^+^ regulatory T cell along with effector cell resistance to suppression at the tissue level seem to play an important role in AIH immunopathogenesis. AIH is predominantly a T lymphocytes driven disease but B lymphocytes are also involved in the immunopathology. Innate immune cells are crucial in the initial onset of disease and their response is followed by adaptive T (Th1, Th17, and cytotoxic T cells) and B cell responses evidenced by liver histology and peripheral blood serology. Standard treatment regimens involving steroid and immunosuppressive medications lead to global immune suppression requiring life-long therapy with many side effects. Biologic therapies have been attempted but duration of remission is short-lived. Future direction of diagnosis and treatment for AIH should be guided by “omics” and the immunology profile of the individual patient and clinicians should aim to deliver personalised medicine for their patients. Cell therapy such as infusion of autologous, antigen-specific, and liver-homing regulatory T cells to restore hepatic immune tolerance may soon be a potential future treatment for AIH patients.

## 1. Background

Autoimmune hepatitis (AIH) is an immune mediated liver disease; however its exact trigger and the underlying mechanism by which AIH develops are still not fully understood although genetic, dietary, and environmental factors seem to play an important role. AIH is characterised biochemically by the presence of elevated serum transaminase levels, histologically by interface hepatitis and the presence of plasma cells, and serologically by increased levels of immunoglobulin G (IgG) with presence of either elevated anti-nuclear antibodies (ANA) or anti-smooth muscle antibodies (ASMA), soluble liver antigen (SLA), and anti-actin or anti-liver-kidney-microsomal antibodies (anti-LKM) [1–3]. Type 1 AIH is more common in adults and is characterised serologically, in around 65% of patients, by the presence of ANA or ASMA with elevated IgG [4]. Type 2 AIH is commonly seen in children and is characterised by the presence of elevated anti-LKM antibodies with elevated IgG [5].

The prevalence of AIH appears to vary between different regions of the world based on ethnic origin. A report from the United Kingdom [6] indicated the incidence to be 3 per 100,000 inhabitants while the point prevalence was estimated to be between 10 and 17 per 100,000 persons in Europe [7].

AIH can affect any age groups [8]. It is more common in females with ratio of 3.5 to 1 [9] and associated with other autoimmune conditions such as coeliac disease, vasculitis, and autoimmune thyroid disease [8, 10]. The clinical severity and outcomes of AIH seem to vary between ethnic populations. African-American patients with AIH tend to be more cirrhotic at index presentation compared to those of northern European descent. Patients of Asian, Arabian, and African origin presented the disease with cholestatic features (both blood tests and histology) at a younger age and they were less likely to respond to standard immunosuppressive treatment [11, 12]. Our current data suggests patients from Asia present more acutely with jaundice at older age and anti-SMA positivity is less frequent compared to Asian patients in UK [13]. These variations in ethnic origin are likely to be due to differences in genetic, dietary, and environmental conditions.

In some patients, AIH can coexist with other autoimmune biliary conditions such as primary biliary cholangitis (PBC) or primary sclerosing cholangitis (PSC), also known as overlap syndrome [14]. Patients with refractory or difficult to treat AIH should be assessed for underlying undiagnosed overlap syndromes.

## 2. Aetiology

The pathogenesis of AIH is a complex process and the exact aetiology is still unknown although genetic and environmental factors play an important role [15]. AIH can occur in genetically predisposed individuals, who are also exposed to environmental factors [16]. Potentially, viral infection or environmental toxin could change hepatocyte epitopes, which could trigger an immune response, possibly via molecular mimicry [16].

### 2.1. Genetic Link in AIH

The major histocompatibility complex (MHC) has been extensively studied and although the precise roles of various MHC alleles are not fully clear, it is believed that specific alleles enhance the autoimmune response by enhancing the immunogenicity of the antigen and thus provoking a strong T cell response. The genetic associations studied most in AIH were Human Leukocyte Antigen (HLA) alleles and the most common HLA loci associated were HLA DR3 and DR4 [18]. HLA DRB1^*∗*^0301 is the principal risk factor for type 1 AIH among Caucasian Northern European adults and is associated with poor prognosis [19, 20]. HLA DRB1^*∗*^0401 is a secondary but independent risk factor in the same population [19, 20] and the presence of the HLA DR4 subtype (DRB1^*∗*^0401) is associated with less severe disease, a lower frequency of relapse, and presentation at an older age compared to patients with DRB3^*∗*^0101 [20]. A recent genome-wide association study of AIH type 1 patients in Netherlands identified the variants of SH2B3 and CARD10 as likely risk factors and these findings support that AIH is a complex genetic condition which overlaps with other immune mediated liver diseases [15].

## 3. Immunopathogenesis

The immunopathogenesis of AIH is complex and remains unsolved. To date, insight from animal studies has been limited due to lack of relevant models. However, a new experimental murine model of AIH involving self-limited adenoviral infection with the hepatic autoantigen formiminotransferase cyclodeaminase (FTCD) closely resembles human AIH histologically and injury can be rescued successfully with steroid therapy [21].

**Figure 1:**
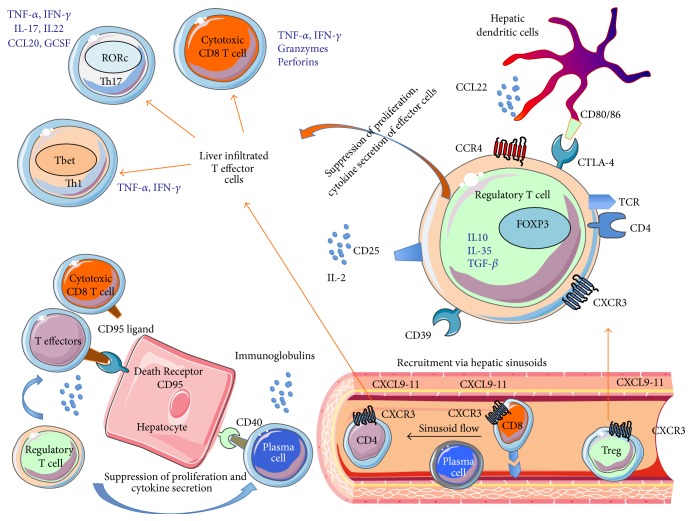
Pathogenesis of autoimmune hepatitis. Both effector T cells (CD4, CD8) and regulatory T cells (Treg) are recruited to inflamed autoimmune hepatitis liver via hepatic sinusoids. T effector cells lead to apoptosis of hepatocytes via CD95 ligand (dead ligand) expressed on them, which binds to CD95 on the hepatocytes. This killing action of T effector cells is regulated by regulatory T cells, which suppress proliferation and cytokine secretion of effector T cells. Plasma cells are also involved in immune-pathogenesis and they secrete immunoglobulin. Liver infiltrated T effector cells consist of Th17, Th1, and cytotoxic T cells. Th1 cells express T bet transcription factor; Th17 cells express transcription factor RORc. Cytotoxic T cells secrete IFN, TNF, granzymes, and perforins. Regulatory T cells (Treg = CD4CD25^high^CD127^low^) express liver tissue homing chemokine receptor CXCR3, which binds to its ligands CXCL9-11 expressed on inflamed hepatic sinusoid, hepatocytes, and bile ducts. Treg also expresses its functional markers CTLA4 (interacting with CD80/CD86 on dendritic cells). Dendritic cells secrete chemokine CCL22, which attracting chemokine receptor CCR4 expressing regulatory T cells. CD39 on the Treg can generate immunosuppressive adenosis from ATP in the hepatic microenvironment. IL-2, which acts on its receptor CD25, is crucial for intrahepatic Treg survival and function. TCR: T cell receptor.

The autoantigen for type 1 AIH is still unknown. Hepatocytes antigen epitope change may play a role in loss of peripheral self-tolerance [22]. In general, acute flare-up of AIH and treatment naïve acute onset AIH is driven by innate immune responses such as natural killer (NK) cells and innate lymphoid cells [23]. Chronic active AIH is characterised by an effector CD4 and CD8 T cell immune response to hepatocytes [24]. Activation of CD4 T cells dominates in the early stages of AIH and is followed by a cytotoxic CD8 T cell response [25]. The precipitating aetiology for flare-up of AIH is still unknown. Thymic derived Treg, defined by surface markers CD4^+^CD25^high^CD127^low^ and transcription factor FOXP3^+^ cells, plays a crucial role in the pathogenesis of AIH [26, 27]. Reduction in frequency and impaired function of CD4^+^CD25^high^ T regulatory cells (Treg) have been reported in the peripheral blood of AIH patients [28, 29]. However, there is a parallel increase in Treg frequency along with effector cell in the inflamed liver tissue [30]. It is possible that Tregs are recruited to the site of immune mediated hepatic injury along with cytotoxic and Th1 effector cells to control the inflammation and thereby causing a reduction in the frequency in the peripheral compartment. Recruitment of cells including Treg to the liver is mediated by the tissue homing chemokine receptor, CXCR3, which interacts with the IFN inducible chemokines CXCL9-11 on hepatic sinusoids, hepatocytes, and biliary epithelial cells [30, 31]. The animal model of concanavalin-A induced T cell mediated hepatitis also suggested the crucial role of Treg and tissue homing receptor CXCR3 (Figure 1) [32, 33].

The duration and severity of hepatitis may be dependent on the balance of Th1, Th17, cytotoxic cells, and regulatory T cells. Flare-up of AIH has been characterised by a parallel increase in frequencies of Treg, effector T cells (Teff), and B cells in the liver [34]. Tregs are enriched in parallel with Teff within livers of patients with untreated AIH-1 with a constant ratio of Treg/Teff while individuals with biochemical remission had higher intrahepatic Treg/Teff and Treg/B cell ratios compared to patients failing to reach remission [25].

Cytokines, interleukin- (IL-) 12, interferon- (IFN-) *γ*, and tumour necrosis factor-alpha (TNF-*α*) along with IL-6 and IL-8 are increased in the diseased liver microenvironment. IL-5 and IL-13, Th2 cytokines are present in the late cirrhotic stage of disease [20, 24, 35]. IL-2 is the crucial survival cytokine, which impacts Treg function and downstream signalling is present at only very low concentrations in normal and diseased liver microenvironment [36].

## 4. Clinical Presentation and Diagnosis

Patients with AIH can present to clinicians with acute, subacute, or chronic stages of disease. Outcomes may differ depending on the course and severity of the disease stage. Initial presentation with acute AIH with jaundice occurs in 20% of patients. Patients with acute severe AIH who presented with deranged synthetic function were found to be more prone to liver transplantation and to have a high incidence of mortality [37]. The majority of patients were asymptomatic or experienced nonspecific flu-like symptoms and some patients were cirrhotic at index diagnosis [16, 38]. Hepatocellular carcinoma (HCC) occurs at a rate of 1.1% per year affecting men and women in equal proportions [39]; accordingly, regular surveillance of HCC is recommended in this cohort of patients [40].

The diagnosis of AIH requires fulfilment of scores based on the criteria that were first proposed by the International Autoimmune Hepatitis Working Group (IAAHG) in 1993 [41] and were updated in 1999 [42]. Although the scoring systems were introduced in 1993 and 1999, they were useful for defining patients in research studies but they were not practical for day-to-day clinical use and hence were revised further in 2008 with the view to provide a simple scoring system that could be used in clinical practice (Table 1).

Liver biopsy is crucial for diagnosis, staging of disease, and exclusion of overlap syndrome as well as for long-term management including withdrawal of immunosuppression for patients with AIH. It is important for initial diagnosis because up to 20% of patients do not have detectable autoantibodies in their serum [1, 43]. The typical findings noted in liver biopsy are interface and lobular hepatitis with a mixed inflammatory infiltrate of lymphocytes and plasma cells [4, 24, 44].

**Figure 2:**
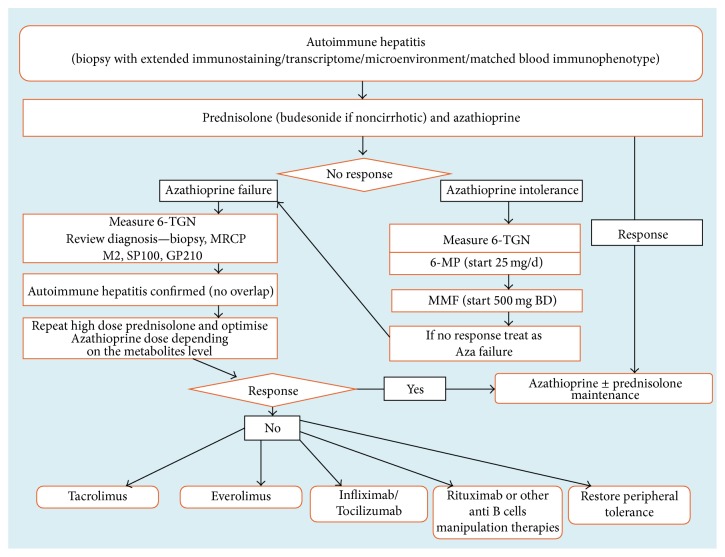
Current stepwise treatment algorithm of autoimmune hepatitis. Aza: azathioprine, 6-TGN: 6-thioguanine, MRCP: magnetic resonance cholangiopancreatography, IL: interleukin, MMF: mycophenolate mofetil, and 6-MP: 6-mercaptopurine.

Future diagnostic tools for AIH might involve a more extensive phenotypic analysis of the peripheral blood, expansion of the liver histology-staining panel, and liver tissue transcriptomic and proteomic analysis including cytokine analysis. It is hoped that, with knowledge on these additional parameters, clinicians will be able to stratify their patients as either “expected treatment responders” or “expected treatment relapsers” and as such determine an appropriate duration of therapy for each of their patients including when to terminate treatment in those expected to regain immune tolerance. With this additional molecular and cell-based information it might also be possible to dissect differential immunological pathways operating in the immune-pathogenesis for personalised treatment options (Figure 2).

### 4.1. Current Standard Care Therapy

Once diagnosis is confirmed by the AIH scoring criteria, treatment should be considered in all patients. There are two phases of treatment in patients with AIH. The initial phase is to induce clinical remission and the second phase to maintain the remission (Table 2). Remission is defined as normalization of serum aminotransferase, a normal level of IgG, and inactive liver histology [24]. Remission is achieved in more than 80% of patients on standard therapy with prednisolone and azathioprine. It is important to note that azathioprine takes 6 to 8 weeks to achieve its immunosuppressive effect. Standard therapy with prednisolone and/or azathioprine leads to remission in 77% of patients with AIH within 6 months of treatment [45].

Histological remission which is indicated by the absence of interface hepatitis lags around 8 months behind biochemical and immunological remission [46]; thus patients with AIH usually require an adequate duration of treatment with steroid followed by a slow and gradual reduction. Premature reduction of steroid is the main reason for failure to achieve remission, which is sometimes wrongly labelled as a “flare-up.” The average duration of treatment to achieve remission is between 18 and 24 months [46]. The common side effects as a result of steroid therapy are cosmetic changes, weight gain, glucose intolerance with risk of developing diabetes or worsening of preexisting diabetes, and osteopenia/osteoporosis. AASLD guidelines recommend screening bone density at diagnosis and at regular intervals afterwards while on steroids [46]. Patients who are on long-term steroids should have calcium and vitamin D3 as part of their medication to prevent deterioration of bone density. Low dose prednisolone is also necessary to prevent the recurrence of AIH following liver transplantation without additional side effects [47]. Incorporating clinical nurse specialist as part of the consultation team for young AIH patients is recommended to improve the compliance in this selected group.

Budesonide is considered an alternative therapy in noncirrhotic AIH patients who experience significant steroid induced side effects since it lacks the systemic side effects due to high first-pass metabolism. Budesonide can be used for both induction and maintenance of remission. The initial dose of budesonide is 9 mg once daily, which can be subsequently reduced to 6 mg and then to 3 mg. A previous double-blind, randomized, controlled, multicenter, phase IIb trial suggested that budesonide is effective in the treatment of noncirrhotic AIH patients with less steroid side effects [48].

In general, azathioprine is well tolerated and necessary to maintain AIH in remission. The common reasons for discontinuation of azathioprine include gastrointestinal side effects, rash, pancreatitis, and myelosuppression. Myelosuppression is more common in those with low levels of the enzyme thiopurine S-methyltransferase (TPMT) and measurement of TPMT is recommended but not compulsory prior to starting azathioprine. Patients with low TPMT levels should be started on low dose azathioprine.

**Figure 3:**
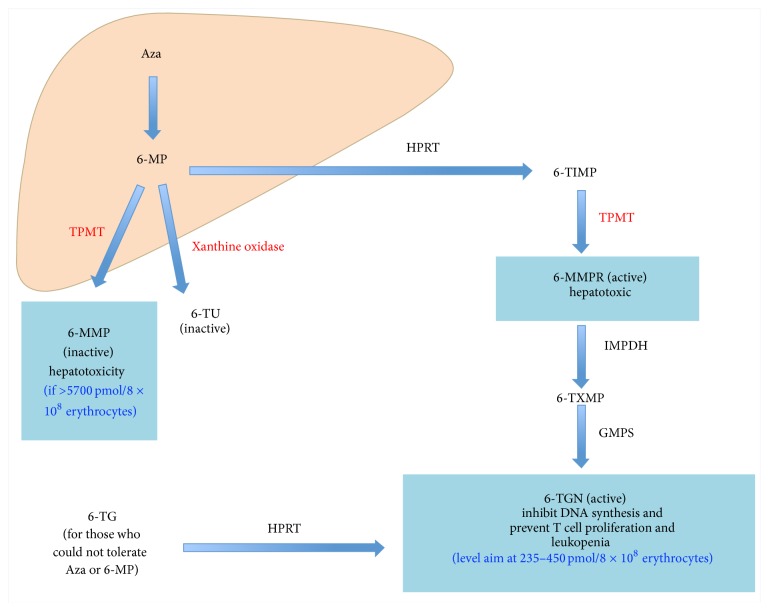
Metabolism of azathioprine. Aza: azathioprine; 6-MP: 6-mercaptopurine; TPMT: thiopurine methyltransferase; 6-MMP: 6-methylmercaptopurine; 6-TU: thiouric acid; 6-TG: 6-thioguanine; HPRT: hypoxanthine phosphoribosyl transferase; 6-TGN: thioguanine nucleotide; GMPS: guanosine monophosphate synthetase; 6-TXMP: 6-thioxanthosine monophosphate; IMPDH: inosine-5′-monophosphate dehydrogenase; 6-MMPR: 6-methyl-mercaptopurine ribonucleotide; 6-TIMP: thioinosine monophosphate.

Azathioprine, a prodrug of mercaptopurine (MP), is converted by nonenzymatic reaction into 6-MP in the liver, which is subsequently converted to 6-MMP (6-methyl mercaptopurine). In erythrocytes, 6-MP is converted to 6-TIMP (6-thioinosine monophosphate) and subsequently to active metabolites 6-TGN (thioguanine nucleotide) [49]. Both 6-TGN and 6-MMP levels can determine whether patients are adherent to the treatment or to optimising the azathioprine dose (Table 2) [50, 51]. Allopurinol can be used to optimise the therapeutic level of azathioprine in some patients with azathioprine related side effects. Measurements of 6-TG (6-Thioguanine) should be taken to establish the therapeutic level of azathioprine, assess patient compliance with azathioprine, and optimise therapy before considering second-line medications. The metabolism of azathioprine is shown in Figure 3.

Most patients will respond to standard treatment but some may have a challenging disease pattern. Such patients are typically described as “*difficult to treat*” and might require alternative or add-on therapy. AIH patients may fall into the “*difficult to treat*” category for multiple reasons. For example, they may be genuine nonresponders despite being compliant; they may be intolerant to the medications as a result of side effects (5–13%); they may have overlap syndrome or a dominant B cell driven pathway of immune-pathogenesis, which is not effectively targeted by the standard therapy. Risk factors for treatment failure include early age of disease onset (<40 years of age), an acute form of presentation, jaundice, or high bilirubin at diagnosis and MELD (Model for End Stage Liver Disease) score of >12 at diagnosis [52].

### 4.2. Alternative Therapies for Difficult to Treat AIH

#### 4.2.1. Mercaptopurine

6-Mercaptopurine (6-MP) is an active metabolite of azathioprine and it can be used interchangeably with azathioprine in patients with underlying AIH. Gastrointestinal related side effects seemed to be more common in azathioprine treated patients than those treated with 6-MP. The role of 6-MP in azathioprine-intolerant AIH patients was recently reported by two tertiary liver transplant centres [53]. The authors proposed that 6-MP appears to be effective and well tolerated as second-line treatment in AIH patients with azathioprine intolerance; however, 6-MP might be ineffective in patients with an insufficient response to azathioprine. Based on the finding, 6-MP may represent an effective alternative treatment option in young women who cannot tolerate azathioprine side effects and who are not suitable for mycophenolate mofetil (MMF) therapy due to teratogenicity side effects (Table 2).

#### 4.2.2. Calcineurin Inhibitors (CNI)

Two commonly used calcineurin inhibitors (CNI) in AIH are cyclosporine and tacrolimus. Cyclosporine binds to cyclophilin and inhibits the phosphatase activity of calcineurin [52]. Cyclosporine therapy is associated with renal dysfunction, hypertension, neurotoxicity, and hypertension; hence, close monitoring is required. Tacrolimus can be used as a salvage therapy in patients with AIH once conventional therapies have failed to achieve remission. Our combined experience with Hamburg transplant unit on tacrolimus suggested that it could be used in compliant patients with difficult to treat AIH in experienced centres. Its use is safe and can improve liver biochemistry and IgG and reduce steroid requirements. Before administration however, it is crucial to ascertain medication compliance; repeat liver biopsy to ascertain ongoing hepatitis is due to AIH etiology and to obtain an updated MRCP (magnetic resonance cholangiopancreatography) to exclude AIH/PSC overlap [54]. Low level should be maintained to prevent nephrotoxicity and metabolic complications (Table 2).

#### 4.2.3. Mycophenolate Mofetil (MMF)

Mycophenolate mofetil (MMF) is a purine antagonist and it has been used selectively as a salvage therapy in AIH. MMF is hydrolysed to mycophenolic acid by liver esterases and acts as a reversible noncompetitive inhibitor of inosine monophosphate dehydrogenase and as such selectively impairs the synthesis of nucleotides based on purines and inhibits new DNA synthesis, impairing the proliferation of activated lymphocytes [55]. The most common side effects are gastrointestinal related symptoms with nausea, diarrhoea and abdominal pain, rash, fatigue, and leukopenia. MMF is contraindicated in pregnancy. A study by Hennes and colleagues suggested that MMF is a suitable alternative for patients who do not tolerate azathioprine and is not likely to be effective if patients had a previous insufficient response to azathioprine therapy (Table 2) [17].

#### 4.2.4. Biologic Therapies

*(A) Anti-TNF-α Agents*. TNF-*α* is a proinflammatory cytokine and it is one of the key cytokines involved in the pathogenesis of AIH. A study by Weiler-Norma and colleagues reported the first series of AIH patients who were treated with infliximab [45]. The study included 11 patients; infliximab was administered at a dose of 5 mg/kg at weeks 0, 2, and 6 followed by every 4 to 8 weeks as per treatment response. Treatment led to reduction of hepatic inflammation as evidenced by a decrease in transaminase levels as well as IgG. Septic episodes were observed in some patients; thus close monitoring is essential. Cases of AIH related to anti-TNF therapy have also been reported [56]; thus infliximab use should be restricted. Understanding the mechanism of pathogenesis for each individual patient and whether the immunopathology of their AIH is driven by the cytokine TNF is crucial before embarking on the therapy. Indeed, prescreening of mycobacterial and blood borne viruses is recommended along with prophylactic antibacterial and antifungal treatment.

*(B) Rituximab*. Rituximab is a genetically engineered, chimeric monoclonal antibody directed against the CD20 antigen on the surface of normal and malignant B cells. Once antigen binds to B cells, it results in lysis and depletion of B cells via complement as well as antibody-mediated cytotoxicity. Burak and colleagues [57] studied Rituximab therapy with a longitudinal follow-up on six patients with biopsy-proven AIH who failed prednisone and azathioprine treatment. The study showed that the infusion was safe with no significant side effects, there was an improvement in liver enzymes and IgG, and reduction in prednisolone dose was achieved in some patients. Stratifying patients at the initial presentation with the aid of new diagnostic tools such as CyTOF (Cytometry Time of Flight) is necessary to guide physicians to the cellular pathway of immunopathogenesis in individual patient to establish whether manipulation of B cells would be advantageous. Potential future therapy would include new B cells manipulation therapies which are in the developmental pathways and currently being applied for other autoimmune diseases.

#### 4.2.5. Sirolimus/Everolimus

Rapamycin/sirolimus is an inhibitor of mammalian target of rapamycin (mTOR) and can selectively induce regulatory T cells. Its beneficial effect has been shown as add-on therapy in treatment resistant patients [58]. Everolimus is a derivative of sirolimus and it has a similar mechanism and efficacy to sirolimus to achieve improvement in transaminase levels [59]. Monitoring of drug level is necessary and these drugs should only be used in experienced centres (Table 2).

### 4.3. Future Therapy

*Regulatory T Cell Therapy*. Autoimmune diseases arise due to a breakdown in peripheral self-immune tolerance. The recent development of GMP (good manufacturing practice) compliant equipment and reagents that can isolate haematological cell populations according to their cell-surface proteins has set the stage for new cell-based therapies. It is hoped that one day cell-based therapies can replace the need for prolonged often life-long global immunosuppression with serious side effects in patients with AIH. Regulatory T cells (Treg) are a subpopulation of CD4 T cells, which is characterised by high expression of the IL-2 receptor alpha chain (CD25) and low expression of IL-7 receptor (CD127). A combined approach to isolate GMP grade CD4^+^CD25^high^CD127^low^ regulatory T cells by either magnetic isolation or cell sorting is now being applied by the investigators who aim to utilize Treg as cell therapy [60]. Tregs are currently accepted as the body's main source of tolerance regulation in the peripheral immune compartments, functioning via cell-contact and soluble factor mediated mechanisms [61] to suppress the destructive proinflammatory and cytolytic activities of immune effector cells.

**Figure 4:**
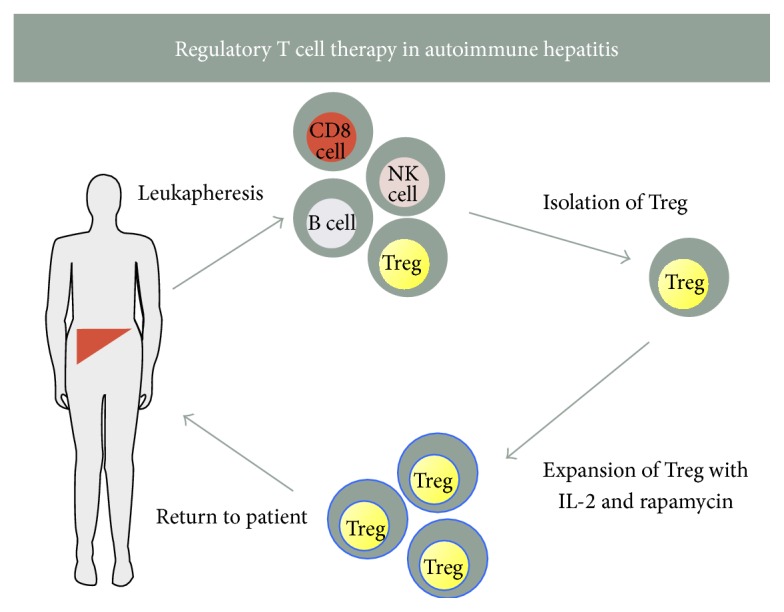
A diagram of regulatory T cell therapy in autoimmune hepatitis. Patients with autoimmune hepatitis undergo leukapheresis followed by isolation of highly pure, autologous, GMP grade, functional CD4 and CD25^high^CD127^low^ regulatory T cells followed by expansion with clinical grade expander beads, IL-2, and rapamycin. These cells can be tested for their purity and stability with flow cytometry and TSDR analysis before infusing back to AIH patients.

The human genetic autoimmune syndrome IPEX (immune dysregulation, polyendocrinopathy, enteropathy, X-linked syndrome), seen among male individuals who lack functional Treg due to mutation in Treg's nuclear transcription factor FOXP3 gene, is a clear indication of the importance of Treg in immune regulation [62]. Moreover, changes in frequency of Treg and functional impairment have been reported in autoimmune conditions [63, 64]. This association between regulatory cell deficiency and inadequate immune tolerance sparked rationale to treat autoimmune diseases by the administering autologous Treg. However, while this strategy proved to be effective in mouse models, its translation into the clinic was initially hampered by the prerequisite requirement to produce GMP grade, sterile, highly pure, and functional Treg in adequate numbers in GMP facilities. It is now feasible to isolate GMP grade CD4 CD25^high^CD127^negative^CD45RA^positive^ clinical grade Treg to switch the immune balance from effector to regulatory arm. Furthermore, with the development of GMP grade anti-CD3/anti-CD28 expansion beads, knowledge of culture conditions that can promote preferential Treg cell outgrowth during cell expansion period by low dose IL-2 and rapamycin supplementation and subsequent assessment of expanded Treg with Treg specific demethylated region by gene analysis facilitate obtaining highly pure and adequate numbers of Treg for clinical application (Figure 4) [65]. The first clinical trial of Treg reported was in the settings of acute and chronic GvHD [66]. Since then the results of further studies in GvHD [66–69] and type 1 diabetes mellitus [70, 71] have reported the safety and feasibility of Treg therapy in humans. In most studies, the evidence suggested potential improvements in clinical, biochemical, and immunological status with Treg therapy. Clinical trials for Treg therapy in AIH patients are yet to be tested and our group is also attempting to conduct a proof of concept investigation of Treg in AIH. Based on our observations of maintenance of expression of the liver tissue homing chemokine receptor CXCR3 in patients with AIH, we anticipate that infused Treg will recruit to the inflamed autoimmune liver tissue [36].

Several studies support the clinical application of Treg in type 1 AIH. Investigators have reported changes in Treg frequencies in AIH patient peripheral bloods [28, 72]; however, there is a recline in functional capacity of liver infiltrated Treg [73]. Reduction in Treg frequency has been shown in patients with primary sclerosing cholangitis [74]. Our group and others have reported that there is an increase in frequency of Tregs along with other effector immune cells in inflamed liver tissue of autoimmune liver diseases [25, 30]. Importantly, we have shown that liver tissue recruitment of Treg is driven by CXCR3 chemokine receptor [30]. It is crucial that following the infusion, tissue resident Tregs remain functional and not plastic to other T cell lineages in the microenvironment. Our group has demonstrated that Treg functional capacity is reduced and lineage is maintained in the microenvironment [73].

Antigen-specific Tregs are anticipated to have greater efficacy and to overcome the possible risk of nonspecific immunosuppression. But until we identify the antigen for type 1 AIH, polyclonal functional GMP Treg in sufficient quantity is the feasible preparation to administer in the treatment of AIH patients. In the future, with advances toward flow sorting and chip sorting technology, isolation of antigen specific Treg subset based on markers including latency-associated peptide (LAP) and glycoprotein-A repetitions predominant (GARP) could be a potential option [75]. Future direction in Treg therapy is heading toward application of expanded CD45RA^+^ Treg cells (either polyclonal or antigen-specific) as these Tregs have an epigenetically stable FOXP3 locus with stable Treg lineage [76]. Analysis of the extent of demethylation of the Treg specific demethylated region (TSDR) may be applicable routinely in the future to verify that the expanded GMP Tregs “are a pure and lineage-stable product” before being infused back into the patients [77].

Once the Tregs are administered intravenously, it is expected that they will migrate to the site of liver inflammation with their homing chemokine receptor CXCR3. The functional capacity of tissue infiltrating Treg is essential to control the ongoing hepatitis by suppressing the effector cells in the inflamed liver. Tregs are dependent upon the cytokine IL-2 for their expansion, survival, and function and owing to their high expression of the high affinity IL-2R alpha chain, CD25, they are sensitive to lower doses of IL-2 compared to effector CD4^+^ T cells, CD8^+^ T cells, and NK/NKT cells [36]. We have observed that peripheral blood Tregs of AIH patients, either in remission or in flare-up state, respond to low dose IL-2 equivocally. The STAT5 signaling pathway is activated to promote a regulatory phenotype and expression of survival factors [36]. Furthermore, we also reported that, in the diseased autoimmune liver, the endogenous IL-2 level is very low [73]. Thus we anticipate that efficacy of Treg therapy may require supportive IL-2 conditioning in the microenvironment. Several clinical studies in HCV-induced vasculitis [78], GvHD [79], and type 1 diabetes have reported that low dose IL-2 therapy is safe, expands Treg frequency, and improves their functional capacity [80, 81]. Accordingly, it is likely that IL-2 might be a crucial cytokine in the treatment of AIH and/or as an adjuvant together with Treg therapy in the future.

Currently Treg therapy is highly expensive due to the clinical reagents, GMP clean room facilities, new technology, and isolation equipment required for generation of the final sterile cell therapy product. Increasing application of GMP grade Treg therapy together with improved understanding of the functional biology of Treg over the coming years will hopefully lead to streamline production of a more economical, practical, clinic based cell therapy. This would facilitate regular application of autologous Treg infusion as novel but standard therapy for patients with AIH in the future offering them freedom from life-long immunosuppression.

## 5. Conclusion

There has been only gradual progression made in the diagnosis and management of autoimmune hepatitis over the last three decades. It is still challenging to tackle the treatment nonresponder, fulminant presenters, and biliary overlap patients. With the advance of genomic, proteomic, metabolomics, and immune profiling, it is prudent to stratify these patients to provide personalized treatment depending on individual multi-OMICs profile. Increasing understanding in immune-pathogenesis of both innate and adaptive responses in AIH patients' blood and importantly in the inflamed liver tissue will pave the way for new therapies.

A novel concept of restoring tolerance with regulatory T cells infusion without requiring life-long global immunosuppression will soon be available in some centres. Adoption of these emerging diagnosis and treatment options is necessary for up-to-date management of patients with AIH.

Exploring causative triggers and antigens and lack of representative animal model still remains a challenge and many investigators are attempting to tackle these aspects to dissect the underlying mechanism. European and global collaborations are necessary to collect a significant number of AIH patients for deeper understanding of immunopathology and to explore new therapies for this rare, immune mediated liver disease.

## Figures and Tables

**Table 1:** Simplified scoring system used for diagnosis of autoimmune hepatitis [17].

Variables	Cut-off	Points
ANA or SMA	≥1; 40	1
	≥1 : 80	2

Or LKM 1	≥1 : 40	2

Or SLA	Positive	1

Ig G	>Upper limit of normal	1
	>1.1x upper limit of normal	2

Liver histology	Compatible with AIH^#^	1
	Typical of AIH^$^	2

Absence of viral hepatitis^*∗∗*^	Yes	2

Exclude other causes of acute liver injury:		
(i) Drug induced liver injury (history)		
(ii) Acute hepatitis A (check HAV IgM)		
(iii) Acute hepatitis E (check HEV IgM)		
(iv) Wilson's disease		

ANA: anti-nuclear antibodies; SMA: anti-smooth-muscle antibodies; LKM1: liver/kidney microsomal antibody type 1; SLA: antisoluble liver antigen.

^*∗∗*^Viral hepatitis: exclusion of viral hepatitis B and hepatitis C. Other viral markers such as *Cytomegalovirus*, Varicella Zoster virus, Epstein Barr virus, and human immunodeficiency virus (HIV) should be excluded.

*Liver Histology*.

^#^Compatible with AIH: chronic hepatitis with lymphocytic infiltration, without typical features.

^$^Typical of AIH: interface hepatitis, emperipolesis, and hepatic rosette formation.

Definite AIH: a cumulative score ≥ 7.

Probable AIH: a cumulative score = 6.

**Table 2:** Overview of standard, alternative, biologic, and future Treg cellular therapies in autoimmune hepatitis.

	Drug	Week 1	Week 2	Weeks 3 to 8	Maintenance therapy	Route	Duration
Monotherapy	Prednisolone (0.5 mg/kg)	40 mg	30 mg	20 mg	Reduction of 5 mg/every 2-3 months	Oral	Once daily

Combined therapy	Prednisolone (OR) Budesonide^*∗∗*^	30 mg 9 mg	20 mg 9 mg	15 mg 6 mg	10 mg ≤6 mg	Oral	Once daily
	With azathioprine^#^ (OR)	1-2 mg/kg (in Europe)				Oral	Once daily
		50 mg/day (in United States)					
		(Dose should be adjusted by 6 TG/6 MMP)					
	6-Mercaptourine	50 mg/day (those who cannot tolerate azathioprine)				Oral	Once daily

Second-line therapies	Tacrolimus^$^	3–5 mg/day (keep level < 6)				Oral	Twice daily
	Mycophenolate mofetil	750–2000 mg (contraindicated in pregnancy)				Oral	Twice daily
		Not effective if patients are previous azathioprine nonresponder					

Biologic therapies	Antitumour necrosis factor (TNF) therapy (infliximab)	5 mg/kg (exclude tuberculosis before treatment)				Intravenous	Once every 2 to 8 weeks
	Anti-CD20 monoclonal antibody (Rituximab) Other B cells manipulating therapies	1000 mg (deep immunophenotype of B and T cells; exclude past hepatitis B infection)				Intravenous	Twice (2 weeks apart)

*Cell therapy* *Autologous regulatory T cell therapy* (Both for induction and maintenance therapy)	Polyclonal or antigen-specific					Intravenous	Trial phase

^*∗∗*^Suitable for noncirrhotic patients with steroid side effects, brittle diabetes mellitus, and osteoporosis.

^#^6-TGN and 6-MMP levels should be monitored for safe therapeutic range. Avoid using with allopurinol. Some centres measure TPMT level prior to starting treatment.

^$^Require therapeutic drug level and renal function monitoring.
